# Mood responses to COVID-19: Implications for family practice in South Africa

**DOI:** 10.4102/safp.v63i1.5285

**Published:** 2021-07-14

**Authors:** Charles van Wijk, Pinky Z. Majola

**Affiliations:** 1 Department of Psychology, Institute for Maritime Medicine, Simon’s Town, South Africa; 2 Department of Global Health, Faculty of Medicine and Health Sciences, Stellenbosch University, Cape Town, South Africa

**Keywords:** COVID-19, lockdown, mental health, mood, fatigue

## Abstract

The effect of coronavirus disease 2019 (COVID-19) on the mood responses of individuals is an important indicator of how society is coping with the pandemic. Characterising mood responses in a South African sample could prepare clinicians for possible presentations of mental health concerns in general practice. This study described mood responses during COVID-19 Alert Level 1. The sample of 641 participants who completed the Brunel Mood State Scale during November 2020 was drawn from primary healthcare and family medicine clinics and practices in Cape Town. Their mood response profile was described and compared with pre-COVID-19 norms. The mood profile represented an *inverse iceberg* profile, with mean scores deviating significantly from pre-COVID-19 norms across all six mood dimensions measured. The *inverse iceberg* profile had been associated with a range of psychopathologies, suggesting an increased risk of psychological disorders. The current profile of mood responses could alert clinicians to the possibility of increased mental health needs of patients. Patient reports of prolonged anxiety and fatigue, particularly when combined with low mood and low vigour, could signal the need for intervention or referral for further mental health support.

## Introduction

### Background

The World Health Organization (WHO) declared the novel coronavirus disease 2019 (COVID-19) a ‘public health emergency of international concern’ on 30 January 2020.^1^ To stem the speed of transmission, significant restrictions were imposed on a large proportion of the world’s population. In South Africa (SA), citizens were expected to reduce social contact and remain home for prolonged periods, colloquially referred to as ‘lockdown’, whilst unemployment skyrocketed as many small businesses were forced to close.^2,3^ South Africa started its ‘hard lockdown’ on 27 March 2020. Restrictions were gradually lifted. By the end of November 2020, over 790 000 people had contracted COVID-19 in SA, with more than 21 500 deaths attributed to it.^4^

Coronavirus disease 2019 and other strains of coronavirus have been shown to impose adverse mental health effects on those who contract the disease,^5,6^ on those placed in precautionary quarantine,^7^ on health caregivers^8^ and on individuals whose daily lives are severely impacted.^9,10^ The WHO described the likely psychosocial effects of the pandemic, with accompanying advice on how to manage mental health,^11^ in the face of predictions of an impending mental health crisis associated with COVID-19.^12^

Locally, two SA studies on the (non-clinical) emotional effect of lockdown found that high numbers of respondents described their emotions during lockdown – by endorsing items from a list – with words such as ‘scared’ (42% – 45%), ‘irritable’ (35% – 39%) and ‘depressed’ (33% – 36%).^13,14^ The South African Depression and Anxiety Group also reported that their call centre volumes had more than doubled since lockdown began.

The effect of COVID-19 on the mood responses of individuals is an important indicator of how society is coping with the pandemic. In particular, narrowly described *mood state profiles* could be useful for indicating long-term outcome risks. Several distinct mood state profiles have been identified, each associated with specific behavioural performance and physical health outcomes.^15,16,17^ For example, the *iceberg* profile, a pattern of mood responses characterised by above average scores for vigour and below average scores for tension, depression, anger, fatigue and confusion has been associated with psychological well-being.^18^ The *inverse iceberg* profile,^19^ characterised by above average scores for tension, depression, anger, fatigue and confusion and below average scores for vigour, has been associated with poorer general mental health,^20^ and an increased risk for a range of specific psychopathologies, including chronic fatigue, post-traumatic stress disorder and eating disorders.^21,22,23^ Recent research on mood state responses during COVID-19 reported an *inverse iceberg* profile in an international sample.^10^ Characterising mood responses in a SA general population sample could prepare clinicians for possible presentations of mental health concerns in general practice.

This study focussed on exploring the mood responses of individuals during COVID-19 Alert Level 1 (instituted from 21 September 2020), a period when restrictions on movement and gathering were reduced, and comparing the observed mood scores with well-established reference norms developed prior to the COVID-19 outbreak.^24,25,26,27,28^ For this study, mood was defined as ‘a set of feelings, ephemeral in nature, varying in intensity and duration, and usually involving more than one emotion’.^29^

### Aim

This study aimed to examine mood responses during COVID-19 Alert Level 1 in SA, to create awareness of adverse mental health expressions that may present themselves during consultations in general practice contexts. To achieve this, the study set two objectives, namely:

- to describe COVID-19 affected *mood state profiles* in a general population sample
- to compare individual mood state responses with local normative data published prior to COVID-19.

## Methods

### Setting and sample

This study used anonymous survey data sourced from primary healthcare and family medicine clinics and practices in Cape Town, during November 2020, prior to the start of the so-called ‘second wave’. The sample comprised 641 adult participants who accessed health services for either non-clinical purposes (e.g. vaccinations, family planning, occupational health certificates), oral health concerns or for minor clinical complaints that could be managed within the clinic or practice without the need for further referral.

Participants were invited to complete the mood response survey anonymously. They could further choose to provide their name and contact number if they desired a follow up, which would consist of a telephone consultation with a psychologist. Probing into mood states posed the risk of exacerbating negative feelings, and care was taken to encourage participants to access the psychological service offered to them by, for example, emphasising its confidentiality and its focus on supporting adaptive coping.

The sample mean age was 37.7 (±12.3, range: 18–70). On request, 14 persons were provided with a follow-up telephone consultation. The sample did not include severely ill patients.

### Measures

Participants reported biographic information (age and gender) and completed the Brunel Mood State Scale (BRUMS).^24^ The BRUMS is a 24-item self-report inventory that measures transient affective mood states.^25,26^ It has been used extensively internationally, amongst others as a marker of mental health.^22,23^ It has been validated across diverse cultures and situational contexts.^15,23,30^ Published South African norms are available,^28^ making it convenient for local use. Good internal consistency, as well as concurrent and criterion validity, has been reported for various versions of the scale, both internationally^25,26^ and locally.^27^

This administration of the BRUMS included six subscales (i.e. tension, depression, anger, vigour, fatigue and confusion), and the standard response timeframe was adapted to specifically refer to the time of COVID-19. Total subscale scores range from 0 to 16 and subscale total scores were transformed into standardised scores for comparison with established tables of normative data for various versions of the scale.^27,28^ It should be noted that the subscales are not diagnostic indicators, but refer to sub-clinical psychological mood states.

### Data analysis

**FIGURE 1:**
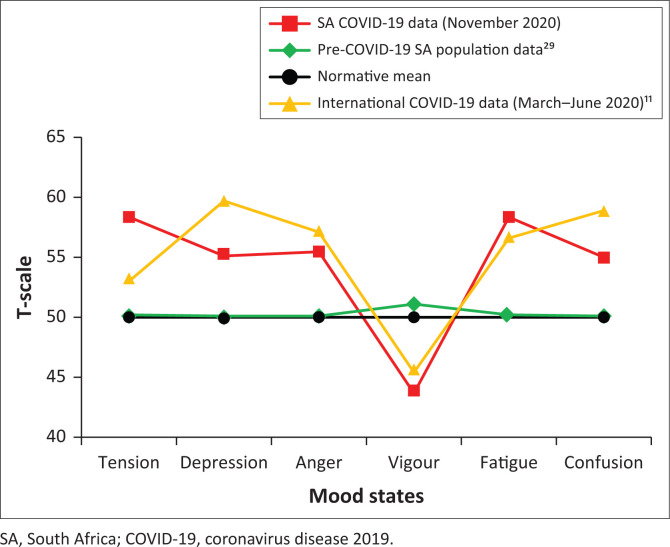
Mean mood profile reported during November 2020.

Data for the month of November 2020 were analysed using statistical software (SPSS version 27). Cases were included if there were no missing values. Subscale totals were transformed to T-scale scores. The standardised data were subjected to the following analyses:

- the collective mood state profile was described using means and visually represented in Figure 1
- the size of deviations of individual mood states from expected population norms were calculated (using *t*-tests) and presented in Table 1
- the effects of age and gender, on any specific mood state, were explored using bivariate correlations and *t*-tests.

**TABLE 1:** Comparison of mean Brunel Mood State Scale *T*-scores versus norms.

Mood dimension	*M*	s.d.	*t*	*d*
Tension	58.36	9.99	21.173*	0.84
Depression	55.11	10.01	12.924*	0.51
Anger	55.46	9.99	13.834*	0.55
Vigour	43.67	10.00	−16.013*	−0.63
Fatigue	58.35	10.00	21.140*	0.84
Confusion	55.00	10.00	12.670*	0.50

*t, t*-test for difference between observed mean and normative mean of 50; *d*, Cohen’s indicator of effect size; s.d., standard deviation; *M*, mean.

* , *p* < 0.001.

### Ethical considerations

The study was voluntary and anonymous, and no written consent was obtained, as completion of the survey was considered implied consent. Ethical approval was obtained from Stellenbosch University’s Health Research Ethics Committee (#N20/11/070_COVID-19).

## Results

The full range of raw scores was observed for all six subscales. After being transformed into T-scores, the mean mood profile of the whole sample, when charted against relevant norms, represented an *inverse iceberg* profile (see Figure 1). The observed mean scores for all mood dimensions differed significantly from the normative mean (i.e. *T* = 50; *p* < 0.001; see Table 1). Effect sizes were large for tension and fatigue scores (*d* ≥ 0.8) and moderate for depression, anger, vigour and confusion scores (*d* ≥ 0.5). This indicated that participants reported significantly raised mood state scores – specifically tension and fatigue – compared with what would historically be expected.

Age was not significantly associated with any mood dimension, whilst women reported higher scores, compared with men, for tension (*p* < 0.01), depression (*p* < 0.05), fatigue (*p* < 0.01) and confusion (*p* < 0.05).

## Discussion

The collective mean mood profile for the sample, compared with normative scores, indicated an *inverse iceberg* profile. This has previously been associated with a range of psychopathologies, which suggests that, in general, this sample may be at increased risk of experiencing some form of psychological disorder. This also aligns with a similar international COVID-19 mood state profile that was temporally related to a survey which reported mental health problems to be at least twice as prevalent as in non-pandemic circumstances.^10,31^

All six mood dimensions deviated significantly from pre-COVID-19 reference norms. In particular, higher tension and fatigue scores were observed than would typically be expected. Whilst this again follows the international inclination, it was noteworthy that the current SA sample reported more tension than depression, as opposed to more depression than tension in the international sample (also see Figure 1).^10^ The observed SA trend is supported by data from two local studies that reported more people subscribing to emotions of being scared or fearful than depressed.^13,14^

Although age did not significantly influence mood responses in this sample, gender differences in mood responses followed the expected direction for this scale, as also seen in other local and international data.^15,16,17,28^

There are several possible explanations for the increase in negative emotional states that was observed. For many individuals, the pandemic brought fear and loss – health fears for self and loved ones and fear of isolation as well as loss of income, social support and a sense of normality.^10^ Furthermore, the need to remain vigilant in complying with non-pharmaceutical measures and the need to manage disruptions in personal, social and occupational routines, may have combined with end-of-year fatigue to create an increased risk of exhaustion and subsequent challenges to effective emotional regulation.

### Limitations

The sample was drawn from individuals accessing healthcare in primary and family medicine settings, during a specified time-period and mood responses may differ across unrelated contexts and fluctuating restriction levels. For example, the comparative studies cited differed from the current SA sample in terms of degree of lockdown restrictions and duration and demographic composition,^10,31^ which all may influence individuals’ life demands or general coping mechanisms. The survey was further conducted in English, potentially excluding participants without reasonable English proficiency, and the findings cannot be generalised across SA society without caution. Further studies that include qualitative designs would be invaluable to better understand the dynamic relationship between mood responses and societal disruption.

### Practical implications

The trend of mood responses observed here should alert clinicians to the possibility of increased mental health needs of patients visiting general medical and dental practices. Based on responses to the BRUMS scale, clinicians could expect to hear patients express their current state of psychological well-being with words such as ‘worried’, ‘anxious’, ‘uncertain’ (about the near future) and ‘tired’, ‘exhausted’, worn-out’. Patient reports of prolonged anxiety and fatigue, particularly when combined with low mood and low vigour, could signal the need for further inquiry into mental health concerns.

## Conclusion

This study examined mood responses in November 2020, during COVID-19 Alert Level 1. It found elevated tension, depression, anger, fatigue and confusion and reduced vigour, representing significant mood disturbance and raising the possibility of an approaching mental health crisis. This underscores the need for awareness of adverse mental health expressions that may present themselves in general practice and other primary healthcare contexts. An important implication of these findings is the need for urgent measures to mitigate the negative impact of the COVID-19 pandemic on mental health.
